# Multiomics profiling of the expression and prognosis of MCMs in endometrial carcinoma

**DOI:** 10.1042/BSR20211719

**Published:** 2021-12-17

**Authors:** Hua Lan, Jing Yuan, Xingyu Chen, Chu Liu, Xiaohui Guo, Xinyu Wang, Jiarui Song, Ke Cao, Songshu Xiao

**Affiliations:** 1 Department of Gynecology and Obstetrics, The Third Xiangya Hospital of Central South University, Changsha, China; 2 Department of Oncology, The Third Xiangya Hospital of Central South University, Changsha, China

**Keywords:** endometrial carcinoma, MCM, methylation, multiomics, mutation

## Abstract

Minichromosome maintenance (MCM) family members are a group of genes involved in regulating DNA replication and cell division and have been identified as oncogenes in various cancer types. Several experimental studies have suggested that MCMs are dysregulated in endometrial carcinoma (EC). However, the expression pattern, clinical value and functions of different MCMs have yet to be analyzed systematically and comprehensively. We analyzed expression, survival rate, DNA alteration, PPT network, GGI network, functional enrichment cancer hallmarks and drug sensitivity of MCMs in patients with EC based on diverse datasets, including Oncomine, GEPIA, Kaplan–Meier Plotter, HPA, Sangerbox and GSCALite databases. The results indicated that most MCM members were increased in EC and showed a prognostic value in survival analysis, which were considerately well in terms of PFS and OS prognostic prediction. Importantly, functional enrichment, PPI network and GGI network suggested that MCMs interact with proteins related to DNA replication and cell division, which may be the mechanism of MCM promote EC progression. Further data mining illustrated that MCMs have broad DNA hypomethylation levels and high levels of copy number aberrations in tumor tissue samples, which may be the mechanism causing the high expression level of MCMs. Moreover, MCM2 can activate or suppress diverse cancer-related pathways and is implicated in EC drug sensitivity. Taking together, our findings illustrate the expression pattern, clinical value and function of MCMs in EC and imply that MCMs are potential targets for precision therapy and new biomarkers for the prognosis of patients with EC.

## Introduction

Endometrial carcinoma (EC) is one of the most lethal malignant tumors of the female reproductive system worldwide [1–3]. According to the pathophysiological characteristics of the patients, as a hormonally regulated disease [4], EC can be divided into two types [5,6]. Type I is estrogen-dependent endometrial adenocarcinoma. Most patients (more than 80%) are of this type. Type II is estrogen-independent, presents most commonly with serous tumors, carcinosarcomas or clear cell tumors, and has an unfavorable prognosis [7–9]. The etiology of EC is mainly related to the history of obesity, diabetes, hypertension and menstrual disorder [10]. However, the accelerated replication cycle of tumor cells provides a cytological basis for the immortalization [11] and invasive metastasis of tumors, the uncontrolled self‐replication and the aggressive metastasis of cancer cells to surrounding tissues [12]. Thus, EC remains prone to recurrence and metastasis [13], resulting in poor prognosis in many patients [14]. Therefore, exploring a novel target for the diagnosis, treatment and prognosis of EC is of great value to distinguish EC patients as high risks, improve the prognosis of patients and provide individual therapeutic strategies for patients with EC.

Unlimited proliferation is a hallmark of cancer [15]. Under physiological conditions, minichromosome maintenance (MCM) family members are involved in the regulation of DNA replication in cells [16,17]. They mainly play a role in the initiation and extension stages of DNA replication. Therefore, it was believed that MCMs are entry points that regulate the cell replication cycle. MCM2-7 participate in the formation of the DNA replication helicase complex, which can interact with histone H3 and H4 through the N-terminal domain [18]. However, once the expression of these genes becomes uncontrolled, they may drive the occurrence and development of tumors. Increasing studies have revealed that MCM family members are dysregulated in different tumor types and then enhance tumor proliferative ability and promoting tumor development [19]. For instance, in breast cancer, MCM2, MCM4 and MCM6 can help distinguish breast cancers with different histological grades. Recent studies have shown that MCMs have important clinical value [20], including diagnostic markers, therapeutic targets and prognosis evaluation [21,22]. MCM2 and MCM3 mRNA expression was up-regulated in EC. The expression of MCM2 and MCM3 in EC is correlated with the strong proliferation ability of tumor cells, suggesting that MCM2 and MCM3 may be involved in the proliferation of EC [23]. Another study on the expression of MCM7 in EC confirmed that the expression of MCM7 significantly correlated with the pathological grade of EC, and the correlation was better than that of Ki67; this indicates that MCM7 may act as a potential therapeutic target and prognostic marker of EC [24].

Most studies on the relationship between MCM family proteins and EC remain at the level of verification of expression and focus on a single member. A systematical biology analysis of tumor genetics has become an effective method for exploring cancer pathogenesis and development. Thus, this paper is to solve this problem. First, we explored the expression patterns, methylation status, relative survival analysis by using Gene Expression Profiling Interactive Analysis (GEPIA) database, Kaplan–Meier Plotter database and the Human Protein Atlas (HPA) database. Then, we used the cBioPortal and UCSC Xena to confirm the gene alteration of MCMs and DNA mythylation. Subsequently, GGI network and PPI network was constructed based on the GeneMANIA database and STRINGS database. GO and KEGG analysis was used to predict biological function and pathway of MCMs in EC. Ultimately, we also investigated the cancer pathway MCMs involved and sensitivity of MCMs to kinds of drugs by the GSCALite database. The present study presents a global view of the relationship among MCM family members and EC from the perspective of multiomics, which provides systematic and comprehensive data for exploring MCMs, indicating that MCMs may be potential drug targets and biomarkers for the diagnosis and prognosis of patients with EC.

## Materials and methods

### Expression level of MCMs in public databases

GEPIA (gepia.cancer-pku.cn/) is a comprehensive and interactive online server that collects cancer microarray data from “The Cancer Genome Atlas” (TCGA) and “Genotype Tissue Expression” (GTEx) [25]. We assessed the transcriptional level of MCM family members and the different expression levels of MCMs in different pathological stages by GEPIA. The transcriptional level of MCMs in different subtypes of EC was analyzed by Oncomine gene expression array datasets [26] (https://www.oncomine.org/resource/login.html). In the present study, the *P* value was set to 0.05, the fold change was set to 2, and the expression of MCM mRNA was evaluated as a significance threshold in different EC subtypes.

### The Kaplan–Meier plotter

The prognostic value of MCMs in ovarian cancer was assessed by using the Kaplan–Meier Plotter database (www.kmplot.com) [27]. The Kaplan–Meier plotter database was constructed based on gene chip and RNA-Seq data from GEO, EGA, TCGA and other public databases and evaluated the impact of 54,675 genes on the survival rate in 21 types of cancers, including breast cancer (6234 cases), ovarian cancer (2190 cases), lung cancer (3452 cases) and gastric cancer (1440 cases). Here, EC data were obtained from 543 samples. The Kaplan–Meier plotter database integrates gene expression information and clinical prognostic value to conduct meta-analysis and research and to discover and verify survival-related molecular markers.

### The Human Protein Atlas

The HPA (www.proteinatlas.org) started to run online in 2003 and is a user-friendly tool that contains a large number of proteins expression levels based on immunohistochemistry assay data [28]. The database analyses protein expression in tissue, protein location in cells, pathological expression of proteins, and RNA expression in the brain and blood. This database was used to validate the post-transcriptional level of MCMs in EC.

### cBioPortal

cBioPortal for cancer genomics (www.cbioportal.org/) is an open access resource that is used to download and explore the visualization and analysis of tumor genomics datasets [29]. It contains DNA copy number data, mRNA and microRNA expression data, nonsynonymous mutations, protein and phosphoprotein level (RPPA) data, DNA methylation data and limited clinical data. The cBioPortal online tool was used to estimate the mutation of MCMs in tumors.

### The GSCALite database analysis

GSCALite (bioinfo.life.hust.edu.cn/web/GSCALite/) provides a platform for genomic cancer analysis [30]. GSCA integrates 10,000 genomic datasets of 33 cancer types from TCGA and more than 750 small molecule drugs from GDSC and CTRP. We analyzed copy number variation, pathway activity and drug sensitivity of MCMs in EC by GSCALite.

### GeneMANIA analysis

GeneMANIA (www.genemania.org) is an online analysis server for searching for genes and gene interactions, protein and genetic interactions, pathways, co-expression, colocalization and protein domain similarity [31]. In the present study, we obtained a list of related genes associated with MCMs from the GeneMANIA database and constructed an interaction network to clarify the relationship between MCMs and their interacting genes.

### STRING analysis

STRING (www.string-db.org) is a prediction server that analyses protein–protein interactions (PPIs) to provide insights into the mechanisms of MCMs in ovarian cancer [32]. In the present study, we used the STRING database to perform a PPI network analysis on MCMs to explore their interactions.

### Transcription factor analysis

UCSC Xena (xena.ucsc.edu) integrates multiple large public repositories, such as TCGA, GETx, ICGC and private datasets [33]. This online platform can analyze and visualize the expression profile, mutation, copy number and clinical information of a single gene or multiple genes. The UCSC Xena tool was used to verify the correlation between the cancer clinical stage and MCM expression level in the same patient cohort and to analyze DNA methylation. After downloading the gene promoter sequence from NCBI online tool, JASPAR (http://jaspar.genereg.net/cgi-bin/jaspar_db.pl), a transcription factor and transcription factor-binding sites prediction database, was used to predict the transcription factors on the promoter region of MCMs and generate a list of potential transcription factors [34]. The matching score threshold was set at 500.

### Sangerbox

Sangerbox (sangerbox.com/) is a free, comprehensive, user-friendly analysis tool for bioinformatics analysis that can carry out a variety of bioinformatics analyses and visualization mapping, and convenient data can be downloaded using the R package [35]. In the present study, pathway and process enrichment analyses of MCMs and genes associated with MCM alterations were conducted via the Sangerbox data analysis platform. The Kyoto Encyclopedia of Genes and Genomes (KEGG) pathways and Gene Ontology (GO) terms for biological process (BP), cellular component (CC) and molecular function (MF) are shown as bubble charts.

### Gene Set Enrichment Analysis (GSEA)

Gene set enrichment analysis (GSEA) (http://software.broadinstitute.org/gsea/index.jsp) (version 4.1.0) is a method to explore the potential role of MCMs in EMT pathway involved in EC [36]. In the present study, the MCMs were categorized as high and low expression in according to the median value from the TCGA database and then analyzed the significant difference in enrichment between the two cohorts.

### miRNA–mRNA interaction

The prediction of the interaction between miRNA and MCMs was performed with online database, GSCALite (http://bioinfo.life.hust.edu.cn/web/GSCALite/), which is also used to investigate the correlation between miRNA and correspondent MCM proteins. Then the Kaplan–Meier plotter database was used to illustrate the correlation between the hub miRNAs expression and disease progression of EC.

## Results

### Expression levels of MCMs in patients with EC

**Figure 1:**
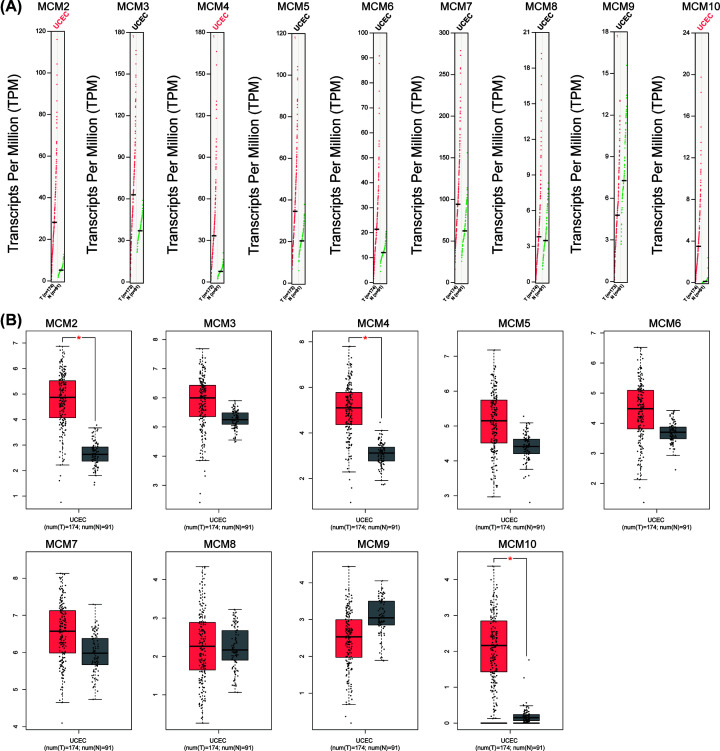
The mRNA expression of MCMs in EC from the GEPIA database

**Figure 2:**
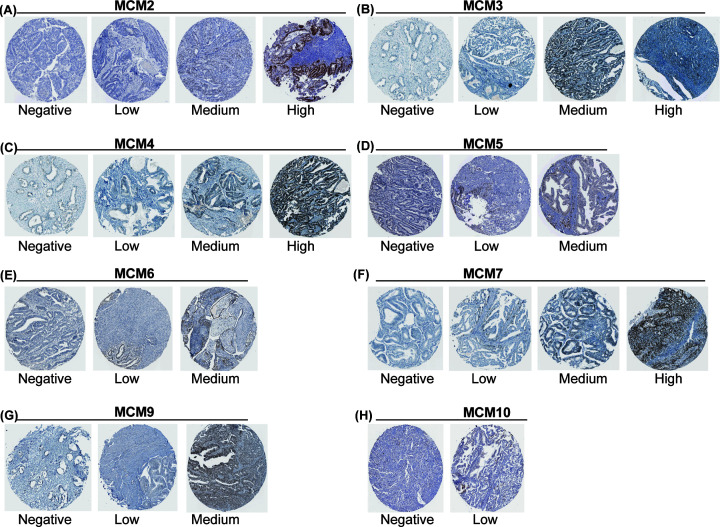
The protein expression of MCMs in EC from the HPA database (IHC)

The GEPIA dataset was used to compare the transcriptional levels of nine MCM proteins in tumors with the corresponding normal tissues of EC. The data showed that the mRNA levels of MCM2, MCM3, MCM4, MCM5, MCM6, MCM7 and MCM10 were significantly increased in patients with EC, while the mRNA level of MCM9 was lower in EC tissue than in normal tissue (Figure 1). In addition, we used tissue microarrays (TMAs) from the HPA database to confirm protein expression in EC tissue and normal tissue, which revealed that extremely elevated MCM2, MCM3, MCM4 and MCM7 expression was detected in 81.8%, 81.8%, 90.9% and 81.8% of the EC specimens, respectively, compared with the normal tissue. MCM5 (40%), MCM6 (45.5%) and MCM9 (25%) were moderately higher in tumor tissues than in normal tissues, and MCM10 was negative in most (91.7%) tumor tissues (Figure 2).

**Figure 3:**
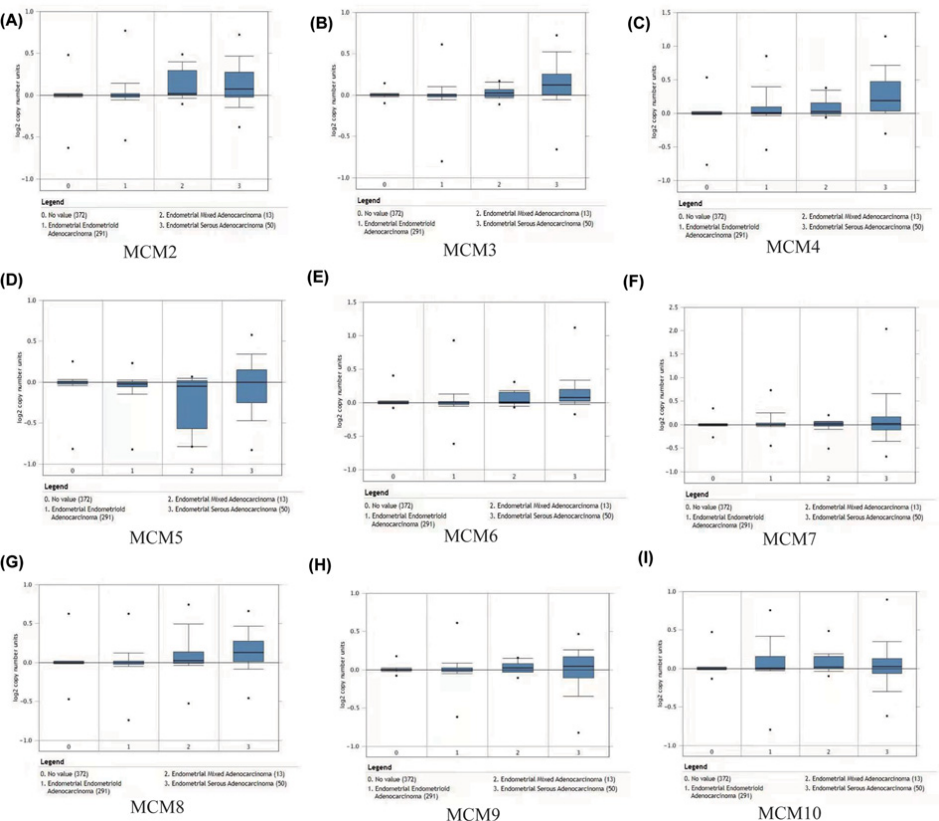
The expression level of MCMs in different EC subtypes

We further examined the expression of MCMs in different EC subtypes, including endometrial endometrioid adenocarcinoma (291), endometrial mixed adenocarcinoma (13) and endometrial serous adenocarcinoma (50) (Figure 3). The results from ONCOMINE showed that the expression of MCM family members in different EC subtypes was of great significance compared with normal control (normal 372). Among them, there were significant statistical differences in endometrial endometrioid adenocarcinoma versus normal in MCM2 (*P*=7.82e-4), MCM3 (*P*=0.016), MCM4 (*P*=6.19e-14), MCM6 (*P*=0.039), MCM7 (*P*=2.20e-7), MCM8 (*P*=0.012), MCM9 (*P*=0.042) and MCM10 (*P*=0.167e-11); endometrial serous adenocarcinoma versus normal in MCM2 (*P*=7.02E-4), MCM3 (*P*=2.56E-4), MCM4 (*P*=7.15E-8), MCM6 (*P*=1.61E-5), MCM8 (*P*=1.09E-4); endometrial mixed adenocarcinoma vs normal in MCM2 (*P*=0.016), MCM4 (*P*=0.022) and MCM6 (*P*=0.043).

Taken together, these results showed that abnormal expression of MCMs is an important biological characteristic in EC patients and may be a promising diagnostic biomarker for EC patients.

### Prognostic value and clinical characteristics of MCMs in patients with EC

**Figure 4:**
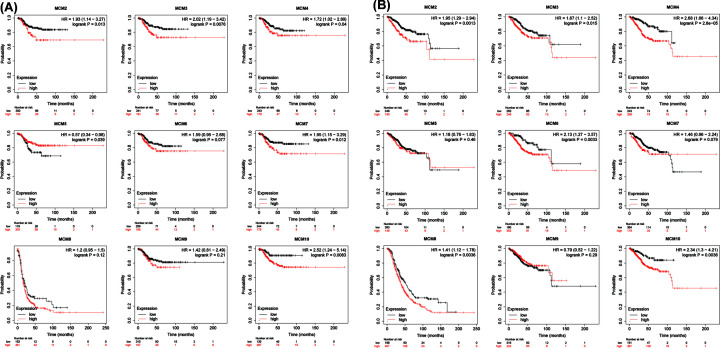
The prognostic value of the mRNA levels of MCMs in EC patients (Kaplan–Meier Plotter)

To further explore the independent prognostic value in EC patients, we used the publicly available website Kaplan–Meier plotter to test the survival rate of different MCMs expression level in patients with EC. The Kaplan–Meier curve and log rank test analyses are shown in Figure 4. Increased MCM2, MCM3, MCM4, MCM7 and MCM10 mRNA levels were significantly associated with poorer progression-free survival (PFS), whereas increased MCM6, MCM8 and MCM9 mRNA levels were correlated with longer PFS (*P*<0.05). The groups with high MCM2, MCM3, MCM4, MCM6, MCM8 and MCM10 mRNA expression were predicted to have worse overall survival (OS), while the others were not related to OS in EC (Figure 4). As shown in Table 1 and Supplementary Tables S1–9, univariate logistic regression analysis based on the data from TCGA further showed that except the MCM9, the up-regulation of MCMs expression in EC was significantly correlated with clinical stage, weight, histological type and high pathological grade. These results indicated that the up-regulation of MCMs expression was significantly associated with poor clinical characteristics, suggesting that EC patients with high levels of MCMs expression are more likely to progress to advanced stages.

**Table 1:** The association between MCMs expression and clinicopathological characteristics in EC (logistic regression, TCGA database)

Characteristic	Clinical stage	Age	Weight	BMI	Histological type	Histologic grade	Age, mean ± SD
MCM2	0.004^*^	0.384	0.003^*^	0.071	<0.001^*^	<0.001^*^	0.524
MCM3	0.056	0.295	<0.001^*^	0.023^*^	<0.001^*^	<0.001^*^	0.262
MCM4	0.021^*^	0.115	0.081	0.652	<0.001^*^	<0.001^*^	0.096
MCM5	0.100	0.474	0.468	0.658	0.101	<0.001^*^	0.391
MCM6	0.017^*^	0.115	0.014^*^	0.324	<0.001^*^	<0.001^*^	0.207
MCM7	0.108	0.684	0.390	0.786	0.006^*^	<0.001^*^	0.905
MCM8	<0.001^*^	<0.001^*^	<0.001^*^	0.055	<0.001^*^	<0.001^*^	0.002^*^
MCM9	0.629	0.175	0.485	0.629	0.456	0.392	0.153
MCM10	<0.001^*^	0.023^*^	<0.001^*^	0.006^*^	<0.001^*^	<0.001^*^	0.056

* *P*<0.05.

### Functional enrichment of MCMs in patients with EC

We then constructed the gene–gene interaction (GGI) network for MCMs including 20 most frequently altered neighboring genes at the gene level. We used GeneMANIA, which illustrates the correlations among shared protein domains, physical interactions, predicted co-expression pathways, colocations and genetic interactions. The GGI network showed that the functions of the 20 most related genes were primarily enriched in DNA replication and G1/S transition of the mitotic cell cycle (Figure 6A).

**Figure 5:**
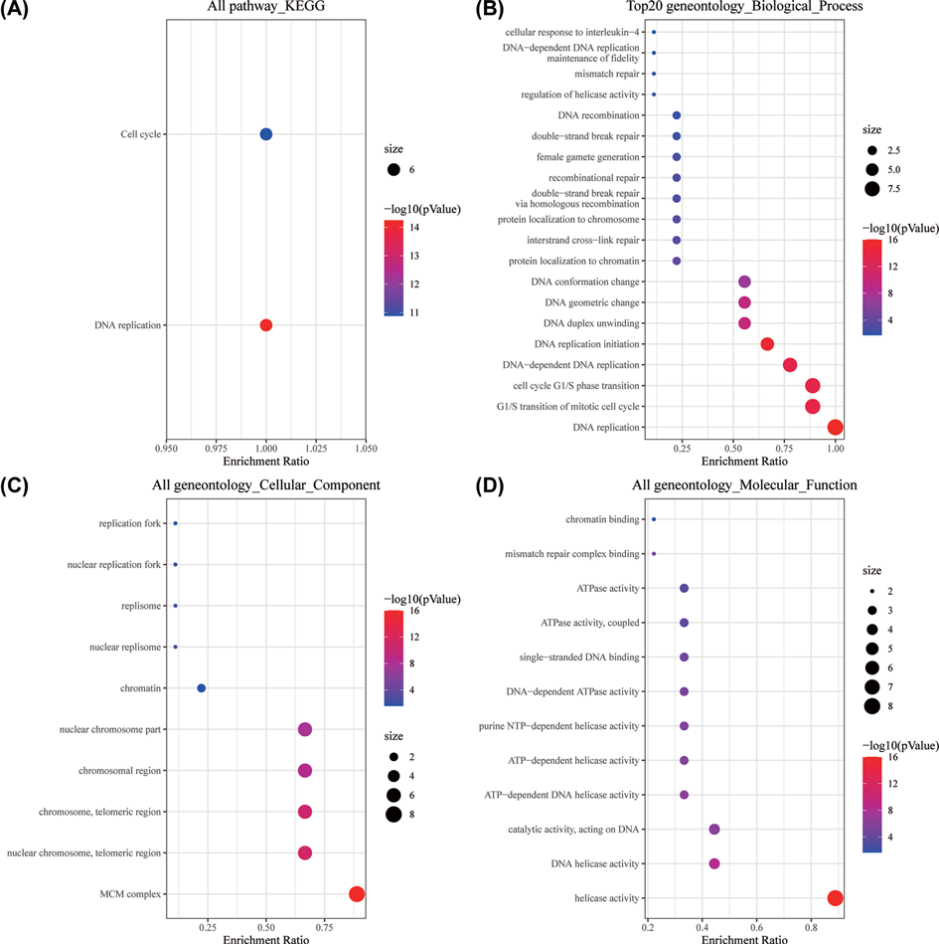
Functional enrichment of MCMs in patients with EC (**A**) KEGG analysis by the Sangerbox online platform. (**B**) The biological process analysis by the Sangerbox online platform. (**C**) The cellular component analysis by the Sangerbox online platform. (**D**) The molecular function analysis by the Sangerbox online platform.

The functions of MCMs and their genes significantly associated with MCM alterations were predicted by KEGG and gene ontology (GO) pathway analyses in the Sangerbox online platform. KEGG analysis revealed that there were only two pathways related to the functions of MCM alterations and neighboring genes. They are involved in the cell cycle and DNA replication, which are also associated with the proliferation of tumor cells (Figure 5A).

**Figure 6:**
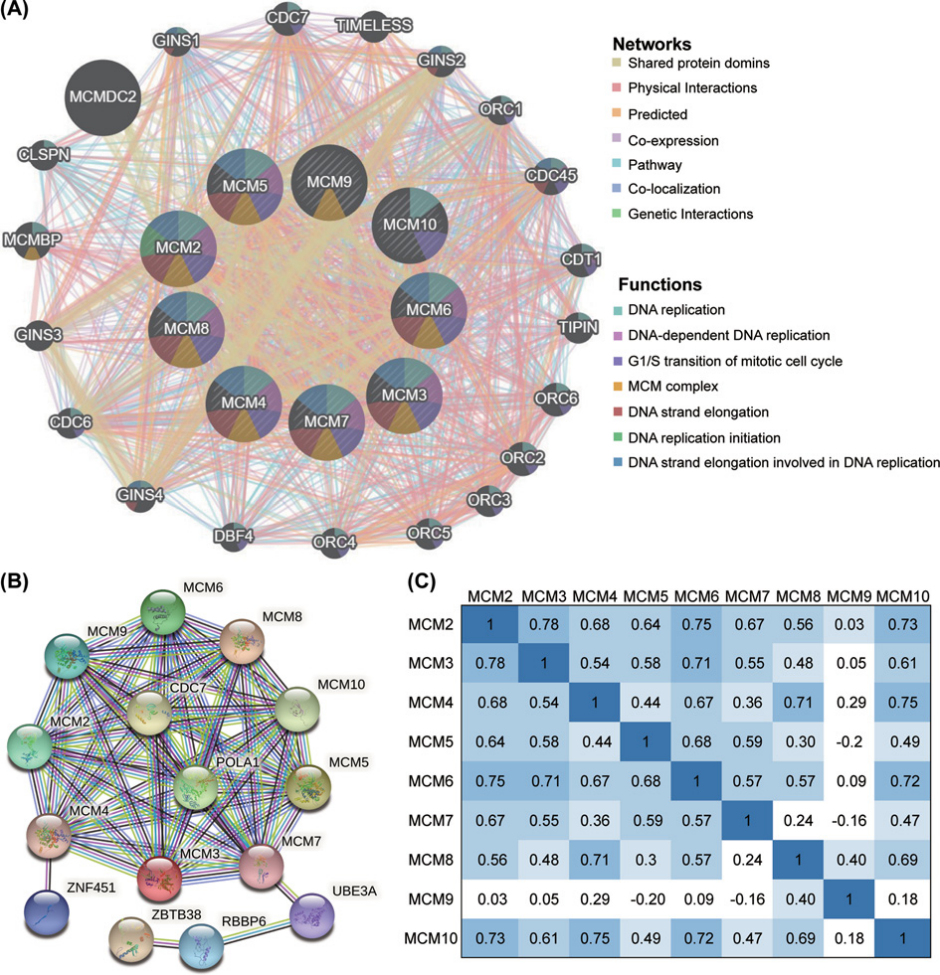
Co-expression and interaction of MCM at the gene and protein levels in carcinoma patients (**A**) The network of MCMs and the 20 most frequently altered neighboring genes by GeneMANIA. (**B**) Protein–protein interaction (PPI) network by the STRING database. (**C**) Relevance between different MCMs in EC by cBioPortal.

Based on the GO biological process analysis, the function of target host genes could be classified into three groups: 20 items in the BP group, 10 items in the CC group and 12 items in the MF group. As presented in detail in Figure 5, genes in BP were primarily associated with DNA replication, G1/S transition of mitotic cell cycle and homologous recombination repair. GO analysis showed that MCM genes exert their functions mainly on DNA helicase activity and ATPase activity. Furthermore, MCM family members are primarily located in the telomeric region and nuclear replisome (Figure 5B–D).

In addition, we explored the function of MCMs and their interacting genes by constructing a protein–protein interaction (PPI) network analysis of MCMs at different transcription levels by the STRING database. The PPI network diagram contains nine MCM proteins and six related proteins. The latter included ZNF451, cell division cycle 7 (Cdc7), POLA1, UBE3A, RBBP6 and ZBTB38 (Figure 6B).

### Possible regulatory mechanisms of MCM expression in EC patients

**Figure 7:**
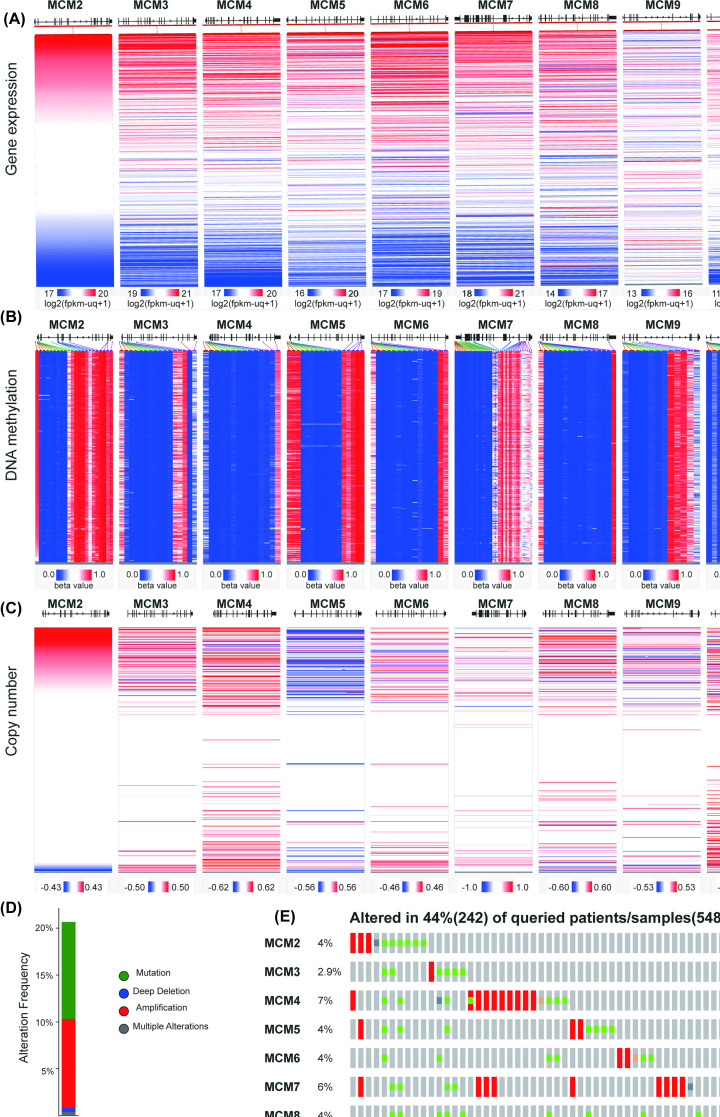
The association between the expression of MCMs and their methylation, copy number and mutation (**A**) Heatmap of MCM mRNA expression by the Xena browser. (**B**) Heatmap of MCM DNA methylation by the Xena browser. (**C**) Heatmap of MCM gene copy number by the Xena browser. (**D** and** E**) MCM gene expression and mutation analysis in EC by cBioPortal.

To elucidate the potential regulatory mechanisms of MCM dysregulation in EC, we examined a heatmap of the expression, DNA methylation and copy number aberration of MCMs from a deep sequencing data cohort using the Xena browser. We observed that compared with normal endometrial tissues, as shown in Figure 7B, the average methylation level in the promoter region of MCMs in EC tissues was significantly decreased, particularly for MCM3, MCM4, MCM6, MCM8 and MCM10. Then, we probed the correlation between the differential gene expression and corresponding copy number aberration profiles in MCM genes (Figure 7A,C). Similarly, MCM genes were widely up-regulated in most tumor samples, which is consistent with the extensive high copy number aberration of MCMs. Taken together, our findings reveal a comprehensive understanding of the methylation and copy number aberration of MCMs. The results indicated that the changes in MCM family protein expression in EC may be primarily caused by the change in DNA methylation level in promoter region and copy number aberration, but the specific mechanism causing the decrease in DNA methylation needs to be explored further.

**Figure 8:**
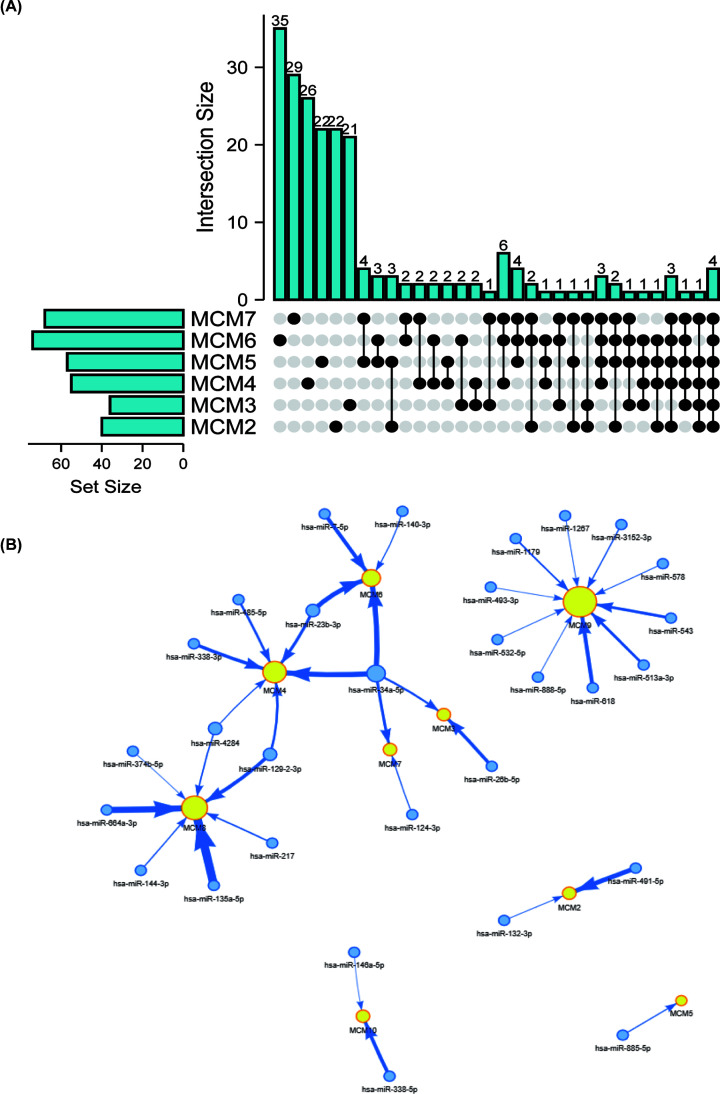
Transcription factor enrichment analysis and miRNA (**A**) A Venn diagram was used to look for candidate transcription factor targeting MCM2-7. (**B**) Regulatory network of miRNA-target mRNA pairs in modular intersection genes. The network contained 30 differentially expressed miRNAs (blue circle) and 9 target MCMs (yellow circle). The width of the arrows indicated the degree of correlation between MCMS and miRNA.

Transcription factors can regulate the gene expression by binding the promotor regions of target gene, which was a significant regulatory mechanism of genes. To investigate the predictive transcription factor binding to the promotor of MCMs, we performed enrichment analysis and generate a list of transcription factors of all MCM proteins (Supplementary Table S10). There was no common transcription factor to all MCMs (Supplementary Figure S1). However, the Venn diagram analyzed by R studio pointed for transcription factors as common to MCM2-7, including MEF2A, MEF2C, RXRG and ZNF384 (Figure 8A). The reason may be the formation of different complexes among the nine members of MCM family. For example, MCM2-7 formed a complex, while MCM8-9 formed another [19].

miRNAs are also a kind of regulated factor that can significantly regulate MCMs expression and function. To investigate the miRNA that were related to MCMs, we filtered the most related miRNAs lists based on GSCALite databases. As shown in Figure 8B, there were only 2 miRNAs regulating MCM2, 2 regulating MCM3, 6 regulating MCM4, 1 regulating MCM5, 4 regulating MCM6, 2 regulating MCM7, 7 regulating MCM8, 10 regulating MCM9 and 2 regulating MCM10. We further investigated the ability of these miRNAs predict prognosis in EC patient by KM plotter. The data showed that 22 of them have significantly correlated to overall survival in EC patients (Supplementary Figure S2). The above results indicated that miRNA-MCM regulatory networks may be important regulatory mechanism for MCM expression and valuable prognostic markers and therapeutic targets for EC patients.

Subsequently, we extracted data from the cBioPortal online tool to analyze the genetic alterations and identify any correlations between genes in EC. Overall, mutations of the MCM gene were altered in 44% (242/548) of EC. The types of alterations included mutations, amplifications, deep deletions and multiple alterations (Figure 7D). The most common genetic alterations were amplifications and missense mutations. Moreover, we analyzed the individual alteration rate for each member of the MCM family. They were 4%, 2.9%, 7%, 4%, 4%, 6%, 4%, 0.8% and 3%, respectively (Figure 7E). Then, we explored the correlation of specific MCMs with each other via cBioPortal, and Pearson’s correction was included. The results indicated that there was a significant positive correlation among MCM gene family members (Figure 6C).

### Cancer hallmarks and drug sensitivity of MCMs in EC

**Figure 9:**
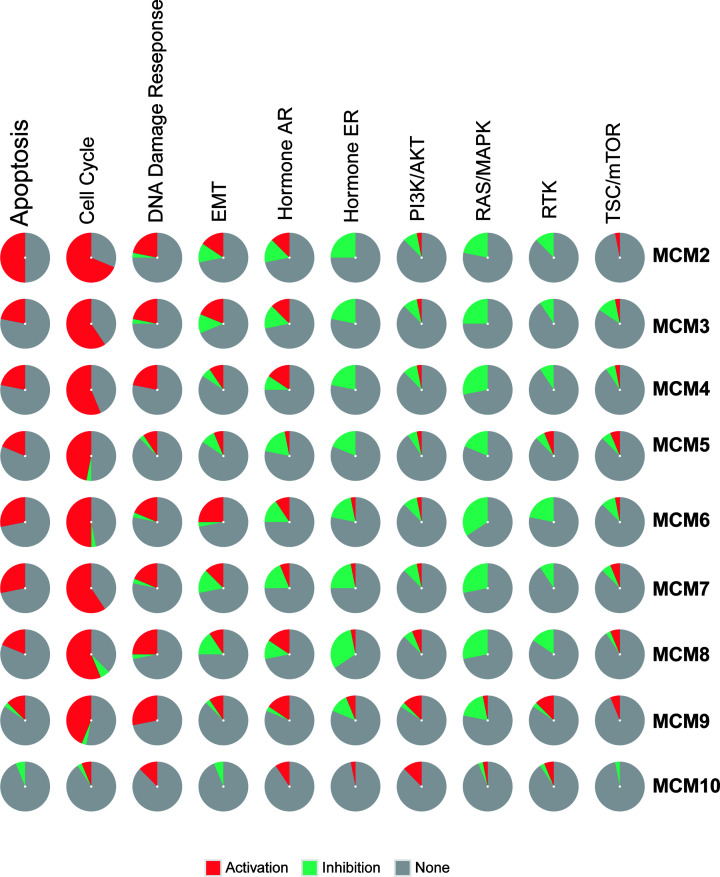
Cancer hallmarks of MCMs in EC Cancer pathway activity analysis of MCMs in EC patients by the GSCALite online tool. Red indicates that pathways may be activated by MCMs in EC, while green indicates that pathways may be inhibited by MCMs.

In common cancer-related pathway enrichment analysis, we observed that the MCM family members can activate apoptosis, cell cycle, DNA damage response, and epithelial–mesenchymal transition (EMT) pathways in EC while inhibiting hormone estrogen receptor (ER), PI3K/AKT, RAS/mitogen-activated protein kinase (RAS/MAPK) and receptor tyrosine kinase (RTK) pathways. These pathways have been widely demonstrated to participate in the proliferation, apoptosis, metastasis and invasion of cancer cells, indicating that the MCM family may be important for the malignant progression of EC (Figure 9). To further understand the potential role of MCMs in EMT, we performed the GSEA analysis to investigate the relationship between MCMs and EMT pathways. The results suggested that EMT pathway-related genes mainly up-regulated in the groups that highly expressed MCM proteins indicating that MCM proteins have a positive relationship with EMT pathway (Supplementary Figure S3).

**Figure 10:**
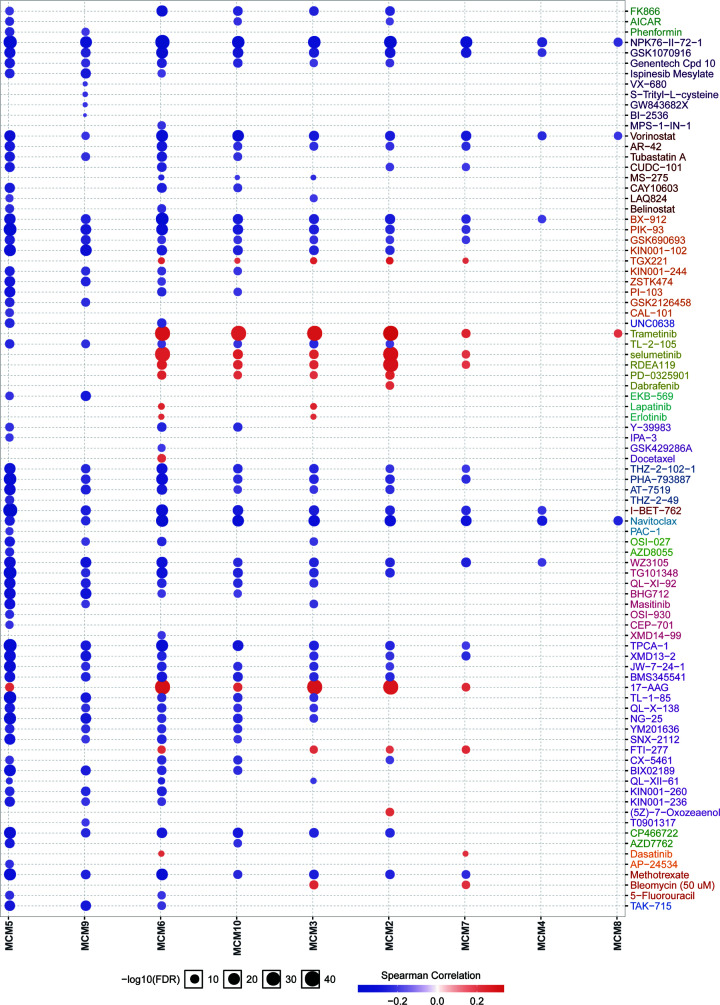
Drug sensitivity of MCMs in EC Drug sensitivity analysis of MCMs in EC patients by the GSCALite online tool. The positive correlation means that high expression of MCM is resistant to the drug.

We next evaluated the role of MCM levels in the sensitivity to small molecule targeted anti-tumor drugs by using the GSCALite online tool. The color and size of the bubbles represent the Spearman correlation and the strength of drug targeting, respectively. These results indicate that the expression level of MCMs was significantly associated with drug sensitivity, suggesting that MCMs may be a potential therapeutic target for EC (Figure 10).

## Discussion

MCM proteins have been reported to be dysregulated in many tumors and are associated with the occurrence and development of cancer. The first MCM protein was isolated from Saccharomyces cerevisiae and was found to be a DNA replication mutant [37]. Further research revealed the standard physiological roles of MCM family proteins, including regulating genomic stability [38], participating in homologous recombination (HR) repair [39] and early embryogenesis [40]. Since MCMs play an important role in regulating DNA replication and homologous recombination repair, MCMs were found to participate in tumor cell proliferation and drug resistance. Although MCMs have been reported to be a potential biomarker for the diagnosis and prognosis of tumors, even as therapeutic targets, further bioinformatics analysis of EC has yet to confirm this. The present study is the first to explore the expression levels, clinical value, biological functions, signaling pathways, genetic variations and DNA methylation of different MCM proteins in EC through bioinformatics analysis.

Among the MCM family, MCM2 is the most investigated in cancers. MCM2 was overexpressed in ovarian cancer. Knockdown of MCM2 increased the sensitivity of ovarian cancer cells to carboplatin via the p53-dependent apoptotic pathway, indicating the therapeutic potential of MCM2 in ovarian cancer [41]. Similarly, MCM2 has been reported to be a potential biomarker of the diagnosis and prognosis of breast cancer [20]. In our study, we obtained data from GEPIA, KM plotter and the Xena browser. The results showed that MCM2 is upregulated in human EC and is related to worse OS and PFS, which is consistent with reports of other cancers.

MCM3 overexpression is an oncogenic event in many types of cancers, including EC, and is correlated with the expression of Ki-67 and estrogen and progesterone receptors [23]. In breast cancer, MCM3 has been reported to be a better biomarker in prognosis than Ki67 [42]. Yang et al. reported that MCM3 is highly expressed in hepatocellular carcinoma and is an independent prognostic factor for patients with HCC. Further study found that MCM3 promotes the development of HCC by activating the NF-κB pathway [43]. In our report, data from a dataset revealed that the expression of MCM3 was higher in EC than in normal tissues and that MCM3 expression was correlated with clinical characteristics.

MCM4 has been reported to be a promising biomarker in the diagnosis and prognosis of several tumors, such as hepatocellular carcinoma [44]. However, little is known about the role and mechanism of MCM4 in cancers. In our study, MCM4 was found to have valuable clinical application because when MCM4 was overexpressed, this expression significantly correlated with poor OS and PFS.

Stockley et al. reported that MCM5 was significantly up-regulated in urine samples from patients with ovarian cancer and EC, indicating the ability of urine MCM5 to discriminate malignant diseases from benign diseases [45]. In our report, we revealed that the expression of MCM5 was higher in EC and is correlated with worse PFS but not OS.

MCM6 is widely considered a biomarker in several cancers, such as hepatocellular carcinoma [46]. Methylation of the MCM6 promoter has been reported to be an important mechanism of proliferation, invasion and migration in breast cancer cells. Moreover, lncRNA LINC00472 can inhibit the expression of MCM6 by recruiting DNA methyltransferases to the MCM6 promoter [47]. In our report, MCM6 maintained a markedly low level of methylation, which is consistent with its high expression, but this expression was not significantly correlated with OS or PFS.

MCM7, one of the unique MCM family members, has been widely reported to be up-regulated in a large number of tumors, including EC [48]. MCM7 was identified as a direct target of PRMT5 in colorectal cancer, and both MCM7 and PRMT5 were up-regulated in CRC tissue and promoted cell proliferation, migration and invasion [49]. In the present study, MCM7 was highly expressed in EC, and this expression correlated with worse PFS but not OS.

MCM8-10 are not involved in the DNA replication complex, but they still play a critical role in cancers, such as gastric cancer [50], cervical cancer [51] and breast cancer [52]. MCM8 and MCM9 were identified to participate in HR repair as MCM8-9 helicase complexes [53]. Thus, an MCM8-9 complex may be a tumor suppressor and potential therapeutic target. As expected, in our report, the expression of MCM8 and MCM9 was not significantly higher in EC and was even lower in the tumor tissue, but MCM10 was highly expressed in tumors. Moreover, the expression of MCM8 and MCM9 was not correlated with PFS, but the expression of MCM10 was related to poor PFS and OS.

As mentioned above, MCMs were enriched in pathways related to DNA replication and the cell cycle and had functions related to DNA helicase activity and DNA repair. Most of the MCMs are associated with CDC7 and POLA1. CDC7, a cell division cycle protein kinase, is a phosphorylase of MCM, and CDC7 is critical for the G1/S transition [54]. DNA polymerase alpha 1 (POLA1) is one of the catalytic subunits of DNA polymerase and plays an essential role in the initiation of DNA replication [55]. These proteins may be important upstream or downstream of MCM in regulating DNA replication. Therefore, further study the mechanisms of MCMs in the occurrence and development of EC provides a new direction for EC research and provides basic data for clinical screening, diagnosis, treatment and prognosis evaluation.

The MCM family members can form different complexes in regulating DNA replication. In previous studies, for example, MCM2-7 can form heterohexamer. In the process of initiate DNA replication, MCM2-7 combined with ORC1-6, Cdc6, as well as the Cdt1, forming ORC–Cdc6–Cdt1–Mcm2-7 complex (OCCM), and then directly binding to DNA replication origins [56,57]. This process has been fully elucidated. MCM8-9 also formed a complex which was a homolog of MCM2-7. Plenty of studies have identified that MCM8-9 function as key molecules in the process of elongation and recombination of DNA [58,59]. It has been reported that MCM8-9 bind to chromatin in late S and G2 which is after most of the DNA synthesis completed. MCM10 is also an essential protein for DNA unwinding during S phase. Warren et al. found that MCM10 had the ability to bind directly to both double-stranded (ds) and single-stranded (ss) DNA though the C-terminal domains (CTD) [60]. Collectively, MCM proteins can directly bind to DNA to regulate gene expression.

With the rapid progression of high-throughput sequencing technology and microarrays, data mining using bioinformatics technology has been undergoing milestone developments [61,62]. In the present study, we obtained multiomic information on MCM family protein members in EC using an online gene analysis tool, and it was correlated with various clinicopathological parameters of EC. Importantly, we provide evidence of the expression patterns, methylation status, relative survival analysis and potential mechanisms of MCMs in patients to further illustrate the effects of MCMs in the etiopathogenesis and pathophysiology of EC.

## Conclusion

From the expression data and prognostic value, MCM2, MCM3, MCM4 and MCM7 should be promising predictors of EC. Functional enrichment indicated that MCMs mainly involved in DNA replication and cell division. DNA methylation, copy number alteration and gene mutation revealed the potential regulatory mechanism of the high expression of MCMs in EC. Then, we extract the data from the GSCALite online tool to investigate the cancer pathway and drug sensitivity of MCM family members in EC. Our study showed that MCMs were closely associated with cancer-related pathways, indicating their critical roles in affecting tumor development. These findings suggest that they may function as potential drug targets in EC and provide new ideas for the treatment of EC. To the best of our knowledge, this is the first systematically analyzing the role of MCM family members in EC from genome, transcriptome and post-translational aspects. The present study had several limitations. First, we failed to test the expression and biological function by cell or animal experimental. Second, the MCMs expression in cancer tissue didn’t test in clinical samples even though we analyzed the IHC results from HPA database. In the future studies, we aim to further assess the expression and prognostic value of MCMs in clinical samples and verify their relationship with the proteins like CDC7 and POLA1 and decipher their biological function *in vivo* and *in vitro*.

## Supplementary Material

Supplementary Figures S1-S3 Tables S1-S10

## Data Availability

The datasets analyzed for this study can be found in on line databases.

## Abbreviations

EMT: epithelial–mesenchymal transition
ER: estrogen receptor
GGI: gene–gene interaction
HR: homologous recombination
MCM: minichromosome maintenance
POLA1: polymerase alpha 1
RTK: receptor tyrosine kinase
