# Supplementary material for: Multiomics profiling of the expression and prognosis of MCMs in endometrial carcinoma
Source: Biosci Rep. 2021 Dec 17;41(12):BSR20211719. doi: 10.1042/BSR20211719 (PMC8685644; doi:10.1042/BSR20211719)
Supplement: Supplementary Figures S1-S3 Tables S1-S10 [file BSR-2021-1719_supp.pdf]

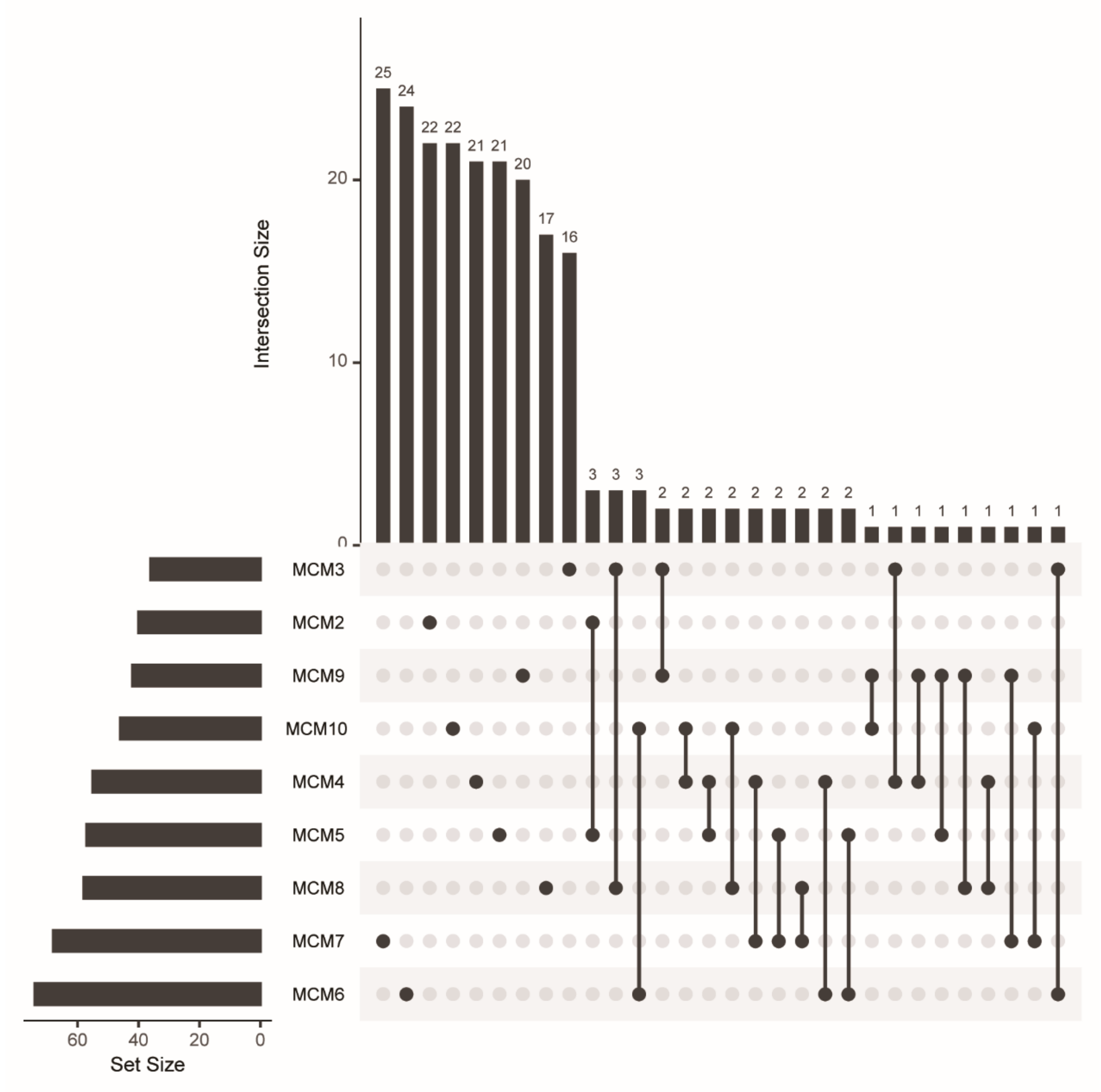

Fig S1 A Venn diagram was used to look for candidate transcription factor targeting MCM2-10.

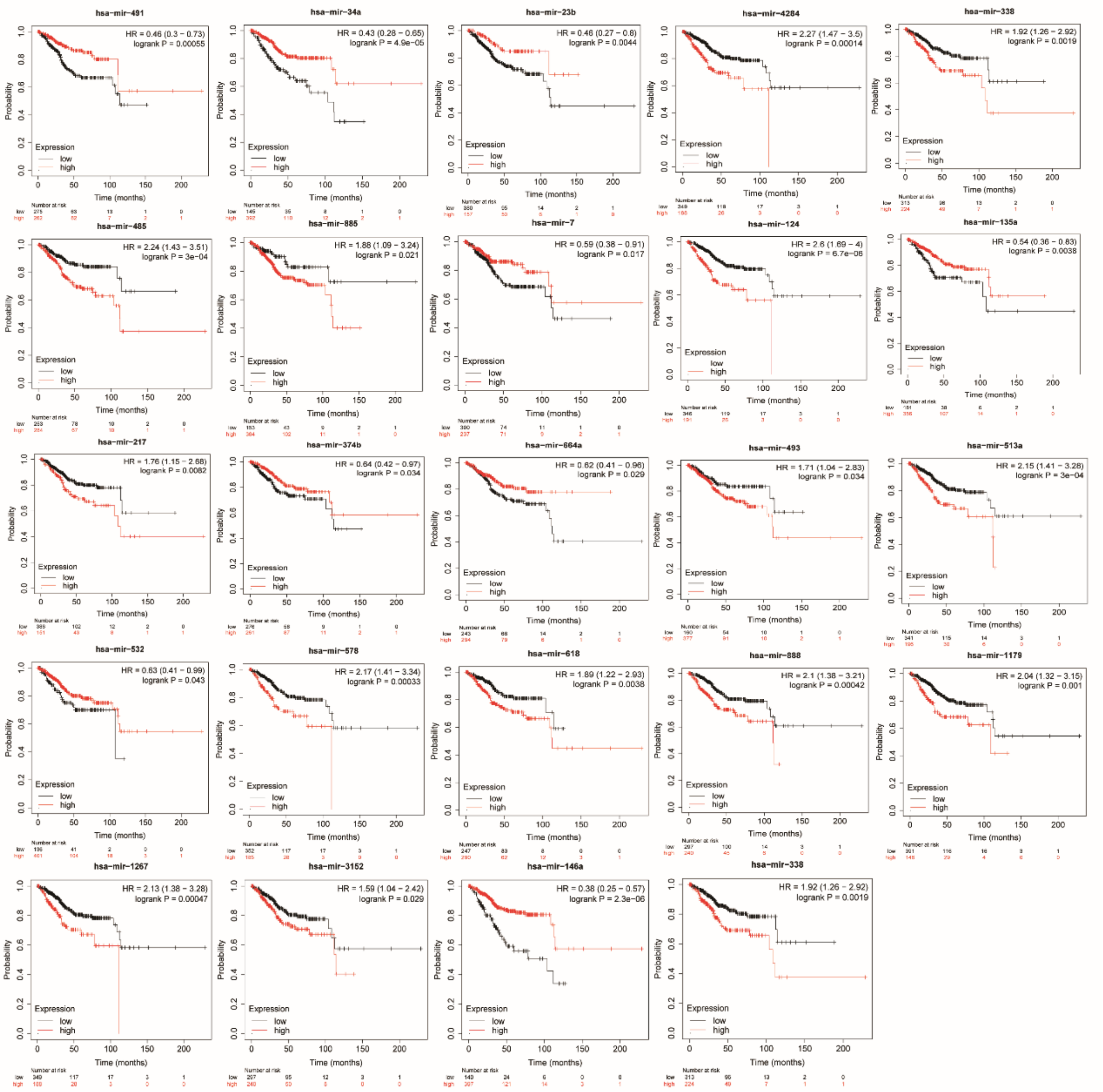

Fig S2 The prognostic value of the expression levels of miRNA in EC patients (Kaplan-Meier Plotter).

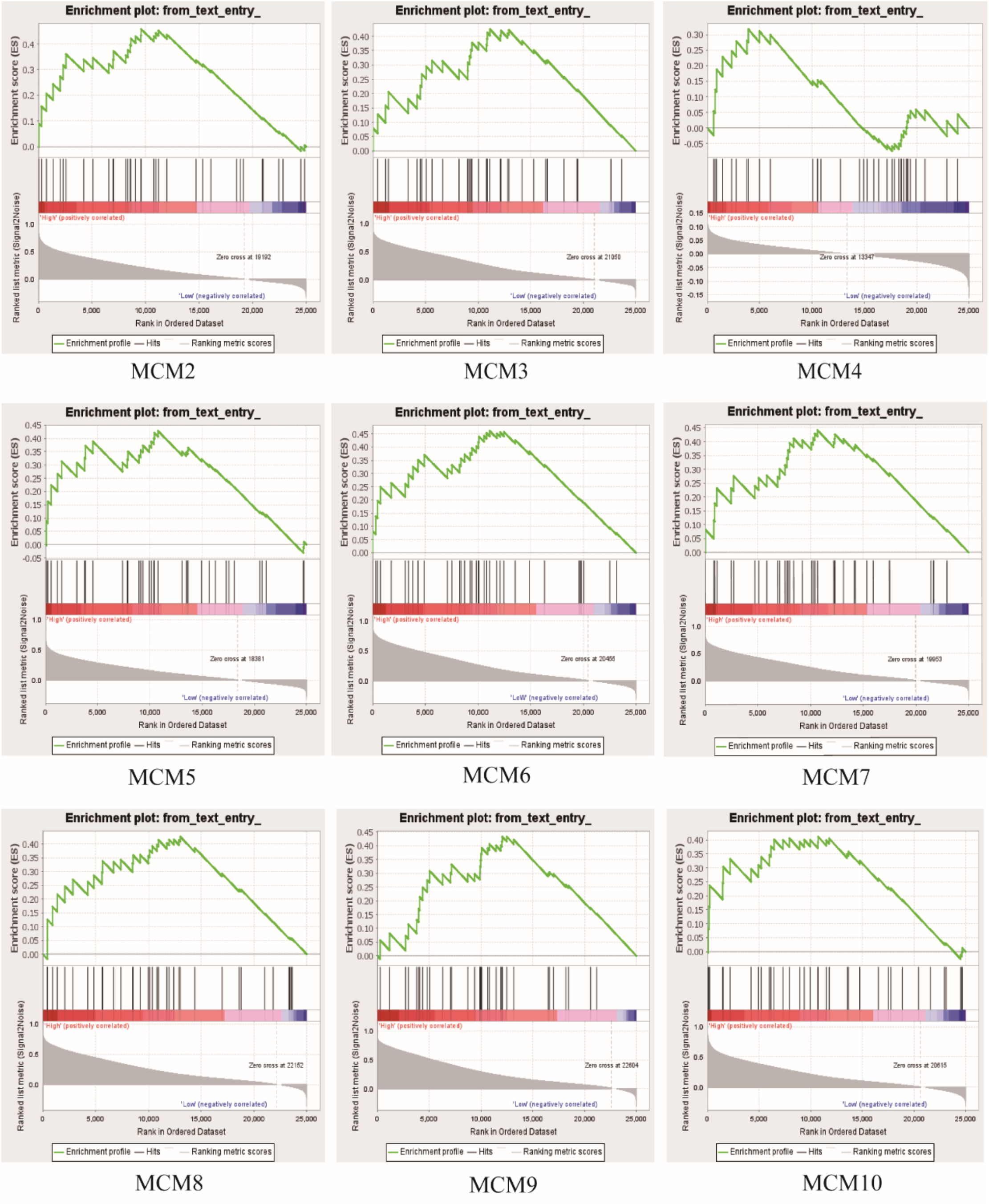

Fig S3 Gene Set Enrichment Analysis (GSEA) validation of EMT signal pathway on MCMs in EC.

Table S1 The association between MCM2 expression and clinicopathological characteristics in EC (logistic regression, TCGA database).

Characteristic	Low expression of MCM2	High expression of MCM2	p
n	276	276	
Clinical stage, n (%)			0.004
Stage I	189 (34.2%)	153 (27.7%)	

Characteristic	Low expression of MCM2	High expression of MCM2	p
Stage II	26 (4.7%)	25 (4.5%)	
Stage III	52 (9.4%)	78 (14.1%)	
Stage IV	9 (1.6%)	20 (3.6%)	
Age, n (%)			0.384
<=60	109 (19.9%)	97 (17.7%)	
>60	167 (30.4%)	176 (32.1%)	
Weight, n (%)			0.003
<=80	104 (19.7%)	139 (26.3%)	
>80	160 (30.3%)	125 (23.7%)	
BMI, n (%)			0.071
<=30	96 (18.5%)	116 (22.4%)	
>30	165 (31.8%)	142 (27.4%)	
Histological type, n (%)			< 0.001
Endometrioid	236 (42.8%)	174 (31.5%)	
Mixed	9 (1.6%)	15 (2.7%)	
Serous	31 (5.6%)	87 (15.8%)	
Histologic grade, n (%)			< 0.001
G1	81 (15%)	17 (3.1%)	
G2	79 (14.6%)	41 (7.6%)	
G3	113 (20.9%)	210 (38.8%)	
Age, mean ± SD	63.78 ± 11.06	64.38 ± 11.09	0.524

Table S2 The association between MCM3 expression and clinicopathological characteristics in EC (logistic regression, TCGA database).

Characteristic	Low expression of MCM3	High expression of MCM3	p
n	276	276	
Clinical stage, n (%)			0.056

Characteristic	Low expression of MCM3	High expression of MCM3	p
Stage I	186 (33.7%)	156 (28.3%)	
Stage II	24 (4.3%)	27 (4.9%)	
Stage III	53 (9.6%)	77 (13.9%)	
Stage IV	13 (2.4%)	16 (2.9%)	
Age, n (%)			0.295
<=60	110 (20%)	96 (17.5%)	
>60	166 (30.2%)	177 (32.2%)	
Weight, n (%)			< 0.001
<=80	99 (18.8%)	144 (27.3%)	
>80	164 (31.1%)	121 (22.9%)	
BMI, n (%)			0.023
<=30	93 (17.9%)	119 (22.9%)	
>30	167 (32.2%)	140 (27%)	
Histological type, n (%)			< 0.001
Endometrioid	237 (42.9%)	173 (31.3%)	
Mixed	9 (1.6%)	15 (2.7%)	
Serous	30 (5.4%)	88 (15.9%)	
Histologic grade, n (%)			< 0.001
G1	78 (14.4%)	20 (3.7%)	
G2	81 (15%)	39 (7.2%)	
G3	115 (21.3%)	208 (38.4%)	
Age, mean ± SD	63.55 ± 11.05	64.61 ± 11.08	0.262

Table S3 The association between MCM4 expression and clinicopathological characteristics in EC (logistic regression, TCGA database).

Characteristic	Low expression of MCM4	High expression of MCM4	p
n	276	276	

Characteristic	Low expression of MCM4	High expression of MCM4	p
Clinical stage, n (%)			0.021
Stage I	188 (34.1%)	154 (27.9%)	
Stage II	23 (4.2%)	28 (5.1%)	
Stage III	55 (10%)	75 (13.6%)	
Stage IV	10 (1.8%)	19 (3.4%)	
Age, n (%)			0.115
<=60	113 (20.6%)	93 (16.9%)	
>60	163 (29.7%)	180 (32.8%)	
Weight, n (%)			0.081
<=80	111 (21%)	132 (25%)	
>80	153 (29%)	132 (25%)	
BMI, n (%)			0.652
<=30	104 (20%)	108 (20.8%)	
>30	158 (30.4%)	149 (28.7%)	
Histological type, n (%)			< 0.001
Endometrioid	227 (41.1%)	183 (33.2%)	
Mixed	11 (2%)	13 (2.4%)	
Serous	38 (6.9%)	80 (14.5%)	
Histologic grade, n (%)			< 0.001
G1	80 (14.8%)	18 (3.3%)	
G2	76 (14%)	44 (8.1%)	
G3	116 (21.4%)	207 (38.3%)	
Age, meidan (IQR)	63 (56, 71)	64 (58, 72)	0.096

Table S4 The association between MCM5 expression and clinicopathological characteristics in EC (logistic regression, TCGA database).

Characteristic	Low expression of MCM5	High expression of MCM5	p
n	276	276	
Clinical stage, n (%)			0.100
Stage I	182 (33%)	160 (29%)	
Stage II	24 (4.3%)	27 (4.9%)	
Stage III	61 (11.1%)	69 (12.5%)	
Stage IV	9 (1.6%)	20 (3.6%)	
Age, n (%)			0.474
<=60	99 (18%)	107 (19.5%)	
>60	177 (32.2%)	166 (30.2%)	
Weight, n (%)			0.468
<=80	115 (21.8%)	128 (24.2%)	
>80	145 (27.5%)	140 (26.5%)	
BMI, n (%)			0.658
<=30	102 (19.7%)	110 (21.2%)	
>30	155 (29.9%)	152 (29.3%)	
Histological type, n (%)			0.101
Endometrioid	216 (39.1%)	194 (35.1%)	
Mixed	10 (1.8%)	14 (2.5%)	
Serous	50 (9.1%)	68 (12.3%)	
Histologic grade, n (%)			< 0.001
G1	65 (12%)	33 (6.1%)	
G2	68 (12.6%)	52 (9.6%)	
G3	141 (26.1%)	182 (33.6%)	
Age, meidan (IQR)	64 (57.75, 72.25)	64 (57, 70)	0.391

Table S5 The association between MCM6 expression and clinicopathological characteristics in EC (logistic regression, TCGA database).

Characteristic	Low expression of MCM6	High expression of MCM6	p
n	276	276	
Clinical stage, n (%)			0.017
Stage I	189 (34.2%)	153 (27.7%)	
Stage II	21 (3.8%)	30 (5.4%)	
Stage III	55 (10%)	75 (13.6%)	
Stage IV	11 (2%)	18 (3.3%)	
Age, n (%)			0.115
<=60	113 (20.6%)	93 (16.9%)	
>60	163 (29.7%)	180 (32.8%)	
Weight, n (%)			0.014
<=80	106 (20.1%)	137 (25.9%)	
>80	156 (29.5%)	129 (24.4%)	
BMI, n (%)			0.324
<=30	101 (19.5%)	111 (21.4%)	
>30	161 (31%)	146 (28.1%)	
Histological type, n (%)			< 0.001
Endometrioid	224 (40.6%)	186 (33.7%)	
Mixed	12 (2.2%)	12 (2.2%)	
Serous	40 (7.2%)	78 (14.1%)	
Histologic grade, n (%)			< 0.001
G1	76 (14%)	22 (4.1%)	
G2	78 (14.4%)	42 (7.8%)	
G3	117 (21.6%)	206 (38.1%)	
Age, meidan (IQR)	63 (56, 71)	65 (58, 71)	0.207

Table S6 The association between MCM7 expression and clinicopathological characteristics in EC (logistic regression, TCGA database).

Characteristic	Low expression of MCM7	High expression of MCM7	p
n	276	276	
Clinical stage, n (%)			0.108
Stage I	185 (33.5%)	157 (28.4%)	
Stage II	22 (4%)	29 (5.3%)	
Stage III	57 (10.3%)	73 (13.2%)	
Stage IV	12 (2.2%)	17 (3.1%)	
Age, n (%)			0.684
<=60	106 (19.3%)	100 (18.2%)	
>60	169 (30.8%)	174 (31.7%)	
Weight, n (%)			0.390
<=80	117 (22.2%)	126 (23.9%)	
>80	149 (28.2%)	136 (25.8%)	
BMI, n (%)			0.786
<=30	105 (20.2%)	107 (20.6%)	
>30	157 (30.3%)	150 (28.9%)	
Histological type, n (%)			0.006
Endometrioid	221 (40%)	189 (34.2%)	
Mixed	11 (2%)	13 (2.4%)	
Serous	44 (8%)	74 (13.4%)	
Histologic grade, n (%)			< 0.001
G1	76 (14%)	22 (4.1%)	
G2	71 (13.1%)	49 (9.1%)	
G3	123 (22.7%)	200 (37%)	
Age, mean ± SD	64.13 ± 11.47	64.02 ± 10.67	0.905

Table S7 The association between MCM8 expression and clinicopathological characteristics in EC (logistic regression, TCGA database).

Characteristic	Low expression of MCM8	High expression of MCM8	p
n	276	276	
Clinical stage, n (%)			< 0.001
Stage I	193 (35%)	149 (27%)	
Stage II	25 (4.5%)	26 (4.7%)	
Stage III	49 (8.9%)	81 (14.7%)	
Stage IV	9 (1.6%)	20 (3.6%)	
Age, n (%)			< 0.001
<=60	124 (22.6%)	82 (14.9%)	
>60	152 (27.7%)	191 (34.8%)	
Weight, n (%)			< 0.001
<=80	99 (18.8%)	144 (27.3%)	
>80	168 (31.8%)	117 (22.2%)	
BMI, n (%)			0.055
<=30	97 (18.7%)	115 (22.2%)	
>30	168 (32.4%)	139 (26.8%)	
Histological type, n (%)			< 0.001
Endometrioid	240 (43.5%)	170 (30.8%)	
Mixed	10 (1.8%)	14 (2.5%)	
Serous	26 (4.7%)	92 (16.7%)	
Histologic grade, n (%)			< 0.001
G1	85 (15.7%)	13 (2.4%)	
G2	81 (15%)	39 (7.2%)	
G3	107 (19.8%)	216 (39.9%)	

Characteristic	Low expression of MCM8	High expression of MCM8	p
Age, meidan (IQR)	62 (56, 71)	66 (59, 72)	0.002

Table S8 The association between MCM9 expression and clinicopathological characteristics in EC (logistic regression, TCGA database).

Characteristic	Low expression of MCM9	High expression of MCM9	p
n	276	276	
Clinical stage, n (%)			0.629
Stage I	168 (30.4%)	174 (31.5%)	
Stage II	23 (4.2%)	28 (5.1%)	
Stage III	71 (12.9%)	59 (10.7%)	
Stage IV	14 (2.5%)	15 (2.7%)	
Age, n (%)			0.175
<=60	111 (20.2%)	95 (17.3%)	
>60	163 (29.7%)	180 (32.8%)	
Weight, n (%)			0.485
<=80	117 (22.2%)	126 (23.9%)	
>80	147 (27.8%)	138 (26.1%)	
BMI, n (%)			0.629
<=30	103 (19.8%)	109 (21%)	
>30	157 (30.3%)	150 (28.9%)	
Histological type, n (%)			0.456
Endometrioid	199 (36.1%)	211 (38.2%)	
Mixed	12 (2.2%)	12 (2.2%)	
Serous	65 (11.8%)	53 (9.6%)	
Histologic grade, n (%)			0.392
G1	55 (10.2%)	43 (7.9%)	
G2	59 (10.9%)	61 (11.3%)	

Characteristic	Low expression of MCM9	High expression of MCM9	p
G3	156 (28.8%)	167 (30.9%)	
Age, meidan (IQR)	63 (56.25, 71)	64 (58, 72)	0.153

Table S9 The association between MCM10 expression and clinicopathological characteristics in EC (logistic regression, TCGA database).

Characteristic	Low expression of MCM10	High expression of MCM10	p
n	276	276	
Clinical stage, n (%)			< 0.001
Stage I	196 (35.5%)	146 (26.4%)	
Stage II	23 (4.2%)	28 (5.1%)	
Stage III	47 (8.5%)	83 (15%)	
Stage IV	10 (1.8%)	19 (3.4%)	
Age, n (%)			0.023
<=60	117 (21.3%)	89 (16.2%)	
>60	159 (29%)	184 (33.5%)	
Weight, n (%)			< 0.001
<=80	98 (18.6%)	145 (27.5%)	
>80	165 (31.2%)	120 (22.7%)	
BMI, n (%)			0.006
<=30	90 (17.3%)	122 (23.5%)	
>30	169 (32.6%)	138 (26.6%)	
Histological type, n (%)			< 0.001
Endometrioid	237 (42.9%)	173 (31.3%)	
Mixed	10 (1.8%)	14 (2.5%)	
Serous	29 (5.3%)	89 (16.1%)	
Histologic grade, n (%)			< 0.001
G1	83 (15.3%)	15 (2.8%)	

Characteristic	Low expression of MCM10	High expression of MCM10	p
G2	82 (15.2%)	38 (7%)	
G3	109 (20.1%)	214 (39.6%)	
Age, meidan (IQR)	63 (57, 71)	65 (57, 72)	0.056

Table S10 A list of transcription factor of all MCM proteins

MCM proteins	Transcription factors
MCM2	RBPJ ELF3 LKZF3 ZBTB7A ELF1 ETV1 ERG GABPA EHF MEF2C MEF2A MEF2D OTX2 ZNF460 ZNF135 ZNF384 ZNF257 KLF5 MAZ RARA MTF1 EBF1 RARB TCF7L1 MAFB YY1 NR2C1 TCFL5 TFAP2E ZNF574 KLF15 RXRG ARNT1 ARNT2 E2F3 E2F4 E2F7 E2F8 PPARG HES1
MCM3	E2F6 E2F8 NFYC PRDM9 MAZ ZNF148 SP5 ZNF701 ZNF81 THAP11 ZNF76 ZNF382 RARB RARG GATA1 SPI1 SPIB ZNF384 HNF1A HNF1B MEF2A MEF2C ZNF354A STAT2 HSF1 HSF2 HSF4 MEOX1 MGA TEAD4 TEAD1 PAX7 POU4F3 LHX3 POU4F1 SOX10
MCM4	PRDM5 SREBF1 ZNF460 LHX3 ZNF652 PITX3 PRDM1 ZFX SIX3 ZNF135 PATZ1 NR2F6 NR1H4 RARG MEF2C MEF2A MEF2D ZNF384 ZNF354A ZNF281 POU5F1 THAP1 ZNF324 ZNF282 DLX2 DLX5 ZBTB26 TFCP2L1 ERF ZIC1 ZNF76 ZNF549 ZIC2 ZIC3 NRF1 ELF2 ELF4 ZNF610 PATZ1FOXD2 RARB E2F7 E2F8 TAL1 FOXJ2 FOXO1 ETV5 GFI1 SP1 SP4 KLF9 SP9 SP3 KLF11 KLF2 KLF6
MCM5	MEF2A MEF2C MEF2D NR5A1 ZNF460 ZNF135 RBPJL ZNF384 SCRT2 PTF1A POU6F2 ARGFX SREBF1 SNAL2 RARB NR2F6 RARG RARA STAT6 ZKSCAN3 MYOG RHOX11 ASCL2 MSC OLIG2 ZNF354A FOXJ3 FOXF1 FOXD3 FOXD2 FOXD1 FOXE1 FOXC2 FOXP2 PRDM5 TFAP2A TFAP2C TFAP2B ZFX ZBTB6 ZNF610 ZNF148 YY2 ELF3 ELF5 E2F2 E2F3 E2F4 E2F7 E2F1 E2F8 RXRB YY1 RXRG PATZ1 ZNF93 SP8
MCM6	ZNF93 FOXD2 PAX2 PAX1 SP3 KLF5 E2F2 NRF1 PRDM9 ZNF148 ZNF281 KLF11 KLF15 KLF16 PATZ1 SP8 SP9 KLF1 KLF10 KLF12 KLF14 KLF7 SP1 SP2 SP4 NPAS4 E2F3 E2F4 E2F7 E2F1 E2F8 ZBTB26 ARID3B NFATC2 RUNX1 ZNF460 ZNF135 ZFX SREBF1 NR2F6 RARG GLI1 GLI2 MEF2A ZNF263 ZNF384 STAT2 STAT1 LRF1 ESR1 NFKB1 NFKB2 MEF2C MEF2D NR1H3 THRA ZFP14 STAT4 LHX1 ALX1 ALX4 LMX1B PTF1A TWIST1 ZBTB18 SPI1 ESRRA NR5A1 NR5A2 RORA FIGLA REL RELA ZNF354A
MCM7	E2F2 E2F1 E2F3 E2F4 E2F7 E2F8 VEZF1 TFEC NFYB ZKSCAN5 NFYA NFYC PBX3 KLF15 KLF7 KLF5 ZBTB26 ZNF675 SP1 SP4 PATZ1 ZNF148 MAZ ETS2 ELK3 RFX1 RFX2 RFX3 RFX5 ZNF417 ZNF460 SREBF1 NR2F6 RARG SP9 MEF2C MEF2A MEF2D NF384 SP3 KLF2 KLF3 KLF6 PRDM9 WT1 KLF4 ZNF281 HOXA6 MEOX2 NEUROG2 ZNF449 ZBTB6 BARX2 FOXJ3 FOXQ1 FOXC2 FOXD3 NR5A1 RARA RARB FOSL1 FOS BNC2 FOSL2 ZNF816 PLAGL1 KLF16 SP8
MCM8	LHX3 PAX7 PARX ARID3B MIX-A PHOX2B GSX1 LHX6 POU6F1 PHOX2A PROP1 ZNF384 ZNF680 ETV2 ZNF460 ZNF135 ZFX SREBF1 NR2F6 RARG MEF2C MEF2A MEF2D HOXA13 TCF4 ASCL1 SNAL1 SNAL3 LRF1 GSC GSC2 PITX3 PRDM9 ZNF75D SP5 KLF7 WT1 PATZ1 ZNF148 MAZ KLF15 E2F6 SP2 ZNF281 ZKSCAN5 ELK1 SP1 SP4 SP3 KLF5 KLF16 SP9 KLF1 KLF10 KLF12 KLF14 ETV6 ERF

MCM9	ZBTB14 SP1 KLF15 KLF1 KLF10 KLF12 KLF14 KLF7 SP2 SP4 ZNF530 ZNF148 PHOX2A LHX2 ALX3 ZNF354A STAT1 ZIM3 STAT2 EBF3 EBF2 NFYA NFYC DMRTA1 DMRTA2 SPIB ZNF701 DUX4 MEIS2 POU2F1 POU3F3 POU3F1 POU3F2 USF1 PRDM1 SPIC ZNF274 POU6F2 NFIL3 ZNF460 DUX SMAD2
MCM10	STAT4 STAT5A STAT1 STAT3 ZBTB32 HOXA13 HOXB13 HOXD13 IRF2 STAT2 ZFP14 FOXC2 ZNF384 FOXD3 ONECUT1 ZNF354A ZNF460 ZNF135 ZFX SIX3 PHOX2B ERF ZNF667 LRF1 NR2E1 IRF7 IRF8 IRF4 ZFP335 MSGN1 FOXB1 IKZF1 THRB NEUROD2 NEUROG2 PTF1A ESRRA NR2F2 RARG CTCF CTCFL SMAD2 ZNF549 GMEB1 SP2 PATZ1
